# Plugging Teeth

**Published:** 1846-12

**Authors:** 


*Plugging Teeth.*—This operation, one of the most important, if not ***the*** most important operation connected with the duties of a dentist, and which is indiscriminately performed by all that bear the name of dentist, from the merest tyro, to such as stand pre-eminent in the profession; is more frequently ill performed, and that too, by those who make no mean pretensions to skill, than any other operation connected with the profession. It might be safely estimated, that not more than one dentist in every hundred, arrives at such a degree of proficiency in this truly difficult branch, as to render the great majority of their operations permanently useful.

For an operator to render justice to himself and the profession, after deciding upon the practicability and permanent success of an operation, and for a correct decision upon which, it requires no little experience and good judgment, there must be no temporising or half way measures taken; every step in the operation must be rendered so complete, that another stroke of the instrument would be one too many. He must not be deterred by the sufferings of his patient, from removing every particle of diseased substance—he must not yield to the popular prejudice of the day, and fail to make application of the file, until the edges of the tooth about the cavity shall insure sufficient strength for the introduction of the plug; and this should be so introduced, that it shall best secure its consolidation in every part of the cavity of the tooth, and the surface of the plug rendered impenetrable to every thing but electricity, and especial care given, that it be completed without leaving irregularities in its union with the edge of the cavity, but when polished, should present a surface as brilliant as a mirror, a union as perfect as if introduced by nature.

Without entering minutely into the means by which the above results are to be effected, we will simply state, that two-thirds of the visible causes of failure that have come under the writer's observation, might be attributed to the manner in which the material used for plugging, was introduced and consolidated in the cavity, and finished upon its surface.

A large number of these failures, we are compelled to believe, results from a want of high-toned professional morals. To plug teeth in general practice, let the excuse be what it may, for a fee not greater than what a *good* practitioner would be willing to obligate himself to pay for the bare material, to plug similar cavities, to say the least of it is savored with dishonesty. If the pecuniary circumstances of a patient require a reduction of fee, do the operation as an act of benevolence, not of profit. To degrade a useful and scientific profession, by failing to devote all the time and talent that such operation demands, should not be countenanced to obtain bread.—*Dental Intelligencer.*

